# Lentiviral-mediated RNA interference targeting stathmin1 gene in human gastric cancer cells inhibits proliferation in vitro and tumor growth in vivo

**DOI:** 10.1186/1479-5876-11-212

**Published:** 2013-09-16

**Authors:** Javed Akhtar, Zhou Wang, Zhi Ping Zhang, Ming Ming Bi

**Affiliations:** 1 Department of Thoracic Surgery, Provincial Hospital Affiliated to Shandong University, 250021, Shandong, China

**Keywords:** Stathmin1, Gastric cancer, Lentivirus, shRNA

## Abstract

**Background:**

Gastric cancer is highly aggressive disease. Despite advances in diagnosis and therapy, the prognosis is still poor. Various genetic and molecular alterations are found in gastric cancer that underlies the malignant transformation of gastric mucosa during the multistep process of gastric cancer pathogenesis. The detailed mechanism of the gastric cancer development remains uncertain. In present study we investigated the potential role of stathmin1 gene in gastric cancer tumorigenesis and examined the usefulness of RNA interference (RNAi) targeting stathmin1 as a form of gastric cancer treatment.

**Methods:**

A lentiviral vector encoding a short hairpin RNA (shRNA) targeted against stathmin1 was constructed and transfected into the packaging cells HEK 293 T and the viral supernatant was collected to transfect MKN-45 cells. The transwell chemotaxis assay and the CCK-8 assay were used to measure migration and proliferation of tumor cells, respectively. Quantitative real-time PCR and western blotting were used to detect the expression levels of stathmin1.

**Results:**

Lentivirus mediated RNAi effectively reduced stathmin1 expression in gastric cells. Significant decreases in stathmin1 mRNA and protein expression were detected in gastric cells carrying lentiviral stathmin-shRNA vector and also significantly inhibited the proliferation, migration in gastric cancer cells and tumorigenicity in Xenograft Animal Models.

**Conclusions:**

Our findings suggest that stathmin1 overexpression is common in gastric cancer and may play a role in its pathogenesis. Lentivirus mediated RNAi effectively reduced stathmin1 expression in gastric cells. In summary, shRNA targeting of stathmin1 can effectively inhibits human gastric cancer cell growth in vivo and may be a potential therapeutic strategy for gastric cancer.

## Background

Gastric cancer is one of the most common cancers worldwide with approximately 989,600 new cases and 738,000 deaths per year, accounting for about 8 percent of new cancers [1]. The worldwide incidence of gastric cancer has declined rapidly over the recent few decades. Despite its recent decline, gastric cancer is the fourth most common cancer and the second leading cause of cancer-related death worldwide [2,3]. It has the highest incidence in China, Japan, Iran, Korea and eastern Asia. The overall prognosis is poor with a 5-year survival rate below 30% in most countries [4]. In China, the decline was less dramatic than other countries; despite an overall decrease in gastric cancer incidence, an increase has been observed in the oldest and the youngest group, and a less remarkable decline has been observed among women than in men [5]. Unraveling the molecular mechanisms underlying gastric carcinogenesis is one of the major challenges in cancer genomics. Malignant transformation of gastric cells is the consequence of a multistep process involving different genetic and epigenetic changes in numerous genes in combination with host genetic background and environmental factors [6].

Stathmin1/oncoprotein 18, also known as STMN-1, is a highly conserved 17 kDa protein. Its function as an important regulatory protein of microtubule dynamics has been well characterized [7]. The protein is conserved in vertebrates and expressed in most tissues [7]. Stathmin performs an important function in regulating rapid microtubule remodeling of the cytoskeleton in response to the cell’s needs. Stathmin’s role in regulation of the cell cycle causes it to be an oncoprotein named oncoprotein 18 (op18). Stathmin can cause uncontrolled cell proliferation when mutated and not functioning properly. Stathmin1 is one of the microtubule-regulating proteins that play a critical role in the assembly and disassembly of the mitotic spindle [8-11]. It is highly expressed in a wide variety of human cancers, including leukemia, breast, prostate, and lung cancer, and provides an attractive target for cancer therapy [12-15]. Our study on esophageal squamous cell cancer revealed that stathmin1 is a predictor of survival in stage IIA esophageal squamous cell carcinoma after surgery [16].

RNA interference (RNAi) is a post-transcriptional process triggered by the introduction of double-stranded RNA (dsRNA) which leads to gene silencing in a sequence-specific manner. The first evidence that dsRNA could achieve efficient gene silencing through RNAi came from studies on the nematode Caenorhabditis elegans. Further analyses in the fruit fly Drosophila melanogaster have contributed greatly toward understanding the biochemical nature of the RNAi pathway [17].

In this study, we have examined the effect of lentiviral mediated knockdown of stathmin gene expression in gastric cancer cells. We showed stathmin1 is overexpressed in gastric cancer derived cell lines MKN-45 and targeting its expression cause decrease in proliferation and migration of MKN-45 cell in vitro and tumor growth in vivo. At present, it is unclear whether stathmin1 is associated with gastric carcinogenesis. The major purpose of this study was therefore to examine the expression of stathmin1 protein and mRNA in gastric cancer-derived cell lines.

## Methods

### Mice, cell lines, culture medium and reagents

BALB/C-nu/nu male mice (weight 18-22 g, 6 weeks old), Human gastric carcinoma cell line MKN-45, Human Embryonic Kidney 293 cell (HEK93T) were obtained from the Shanghai Tumor Institution. Classical Liquid Media Dulbecco’s Modified Eagles Medium (DMEM), High Glucose RPMI Media 1640 was purchased from HyClone (Thermo Scientific). Fetal bovine serum (FBS) was purchased from Gibco (Invitrogen Co., USA). Dimethyl sulfoxide (DMSO) was purchased from Sigma-Aldrich (St Louis, Missouri, USA). Rabbit Anti-STMN-1 Polyclonal antibody and HRP-conjugated secondary antibodies were purchased from Abcam (Cambridge, MA).

All experimental procedures using animals in the present study had received prior approval by the Institutional Animal Care and Use Committee of Shandong University under Contract 2011–0015.

### Cell culture

MKN-45 cell lines were grown in RPMI 1640 medium supplemented with 10% fetal bovine serum (FBS) and 100 units/ml penicillin, and 100 mg/ml streptomycin (Gibco). The cells were grown at 37°C in a humidified atmosphere containing 5% CO2. Stock cultures of each cell line were routinely sub-cultured at least once a week and the medium was changed every 2–3 days.

### Lentivirus-mediated short hairpin RNA (shRNA) knockdown of gene expression

pGIPZ-lentiviral shRNAmir vectors targeting human stathmin1 gene and Non-silencing pGIPZ control vector were purchased from Open Biosystems (Thermo Fisher Scientific, Inc.). pGIPZ non-silencing control vector for use as expression control to generate non-silencing lentiviral stock to optimize expression conditions in the mammalian cell line of interest. pGIPZ cloning vector containing Turbo GFP reporter and also elements required to allow packaging of the expression construct into virions (i.e. 5′ and 3′ LTRs and Ψ packaging signal). pGIPZ vector also expresses a puromycin-resistant gene.

The sequences of STMN1 shRNA are as following: *5’-TTATTAGCTTCCATTTTGT-3’* and *5’-TTATTAACCATTCAAGTCC-3’.* Lentiviral shRNA was produced by Co-transfection of the Trans-Lentiviral packaging mix with a shRNA transfer vector into HEK 293T packaging cells (OpenBiosystems). For cell infection, viral supernatants were supplemented with 6 μg/mL polybrene and incubated with cells for 24 hours. MKN-45 cells were transduced by the lentiviral particles followed by puromycin selection (1 μg/mL) for 10 days. The cells stably expressing shRNA were maintained in puromycin (0.2 μg/mL).

### RNA extraction and qRT-PCR

Total RNA extraction was performed using Trizol reagent (Invitrogen) according to the manufacturer’s instruction. RNA concentration was measured by Nano Drop 1000 (Thermo Fisher Scientific). One microgram of total RNA extracted from the cells was subjected to reverse Transcription (RT). Verso cDNA Ki (Thermo Scientific) was used for cDNA synthesis. Real-time RT-PCR was used to quantify the expression level of Stmn1 gene in gastric cancer cell lines MKN-45 using ABI 7300 real-time PCR thermal cycle instrument (ABI, USA), according to the supplied protocol. Amplification conditions were as follows: Reverse-transcription reaction: 42°C, 30 minutes per cycle. PCR cycling conditions were as follows: Enzyme activation 95°C 15 minutes per cycle, denaturation 95°C at 15 seconds per 40 cycles and Annealing/Extension at 60°C for 60 seconds.

A Real-time PCR reaction was performed using the Solaris qPCR Gene Expression Master Mix with LOW ROX premixed and 1 μL of total cDNA in each well, Stathmin specific primers were as follows: *(F, AGAATACACTGCCTGTCGCTTG; R, AGGCACGCTTCTCCAGTT).* The relative expression levels were normalized to expression of endogenous Beta-Actin. Primers: (*F, TGGAGAAAATCTGGCACCAC; R, GGTCTCAAACATGATCTGG).*

### Protein extraction and Western blotting

For whole-cell protein extraction, cells were washed with cold PBS and subsequently lysed in cold RIPA lysis buffer (50 mM Tris–HCl, pH 7.4, 150 mM NaCl, 1 mM dithiothreitol [DTT], 0.25% sodium deoxycholate, 0.1% NP-40) containing 1 mM phenylmethysulfonyl fluoride (PMSF), 50 mM sodiumpyrophosphate, 1 mM Na3VO4, 1 mM NaF, 5 mM EDTA, 5 mM EGTA, and protease inhibitors cocktail (Roche). Cell lysis was performed on ice for 30 minutes. Clear protein extracts were obtained by centrifugation for 30 minutes at 4°C. Protein concentrations were determined by the method of Bradford using the Bio-Rad protein assay reagent (Bio-Rad) and 20-40 mg of protein mixed with loading buffer was loaded per lane, separated by 12% SDS-polyacrylamide gel electrophoresis (SDS-PAGE). Proteins were transferred to PVDF membrane filters (Millipore, USA). Nonspecific binding was blocked by incubation in phosphate-buffered saline (PBS) containing 0.1% Tween 20 (PBS-T) and 5% skim milk. PVDF membranes were blocked with 5% dry milk for one hour at 4°C. Membranes were incubated in STMN1 primary antibody (1:1000) overnight at 4°C. The membranes were then incubated with the corresponding secondary antibody (1:2000, horseradish peroxidase-conjugated anti-rabbit) in TBST-5% nonfat milk for 1 hour at room temperature and the immunoreactive bands were visualized using EZ ECL Chemiluminescence Detection Kit for HRP (Biological Industries Ltd, Israel). Images were acquired using the LAS3000 Imager (Fujifilm). Membranes were re-probed for Beta-Actin as a loading control.

### Cell proliferation assay

Cell Counting Kit-8 (CCK-8; Dojindo) was used in cell proliferation assay. 3000 viable cells per well into 96-well tissue culture plates in a final volume of 100 μl. Every 24 hours, a plate was subjected to assay by adding 10 μl of CCK-8 solution to each well, and the plate was further incubated for 4 hours at 37°C. The absorbance at 450 nm was measured with a micro plate reader. The experiment was performed in eight replicates.

### Migration and invasion assays

For trans-well migration assay, 50,000 cells were added to upper chamber in serum free media and migration at 37°C towards 10% FBS containing growth media was determined either after 24 hours or 48 hrs. Cells migrated through the membrane were fixed, stained with H&E (Sigma) and counted under light microscope. For invasion assay, lower chambers of matrigel coated invasion plates were coated with 10 mg/ml fibronectin overnight at 4°C and cells invading through matrigel were fixed and stained after 48 hours.

### Immunocytochemistry

Stably transfected MKN-45 cells were seeded into 4-chambered glass slides (Nunc Lab-Tek Chamber Slide System). Cells were then incubated overnight. After 24 hours, cells were rinsed with PBS, fixed with 3.7% w/v paraformaldehyde (Sigma), rinsed with PBS, and permeablized in 0.5% Triton X-100 (Sigma). Nonspecific immunoglobulin binding was blocked with 5% normal goat serum and 0.5% NP-40 (Sigma). Primary antibodies recognizing with stathmin1 (abicam) were diluted 1:100 in blocking solution. After incubation with primary antibody, cells were rinsed with 0.05% Tween-20 (Bio-Rad) in PBS, and then incubated with secondary antibody for 1 h at RT. Stain with 3,3′-Diaminobenzidine (DAB) and observed under light microscope.

### Immunofluorescence

To examine the protein expression of stathmin1 we performed immunofluorescence analysis. Briefly, the cells were washed with PBS and fixed in 4% paraformaldehyde for 10 minutes at 37°C followed by absolute methanol for 10 minutes at 4°C and blocked in PBS containing 1% skimmed milk for 10 minutes. The samples were then incubated with affinity-purified Rabbit Anti-Human Stathmin 1 Polyclonal Antibody (Abcam,USA) at a dilution of 1:100 for 2 hours, rinsed twice with PBS, and incubated with goat anti-rabbit IgG Alexa Fluor 488 (Molecular Probes) for 1 hour.

### In vivo studies of gastric cancer xenograft tumor models in nude mice

Six-week-old male BALB/c nude mice were housed in a temperature-controlled, pathogen-free animal facility with 12-hour light and dark cycles. Mice were injected subcutaneously into bilateral flanks with untransfected cells, or transfected with Non-silencing shRNA and stathmin shRNA (2× 10^6^ cells in 200 μl PBS) to establish tumors. Tumor mass (xenograft) volume was measured every week from week 3 to week 7. After 7 week mice were sacrificed, and tumors were harvested.

### Statistical analysis

For comparison of more than three groups, we used one-way analysis of variance, followed by Tukey’s multiple comparison-values < 0.05 were considered statistically significant. One-way analysis of variance (ANOVA), followed by the LSD post hoc test was used to compare mean differences in 2 or more groups. All statistical analysis was performed by using IBM SPSS version 20.0.

## Results

### Stahmin1/Op18 expression in MKN-45 cell lines

**Figure 1:**
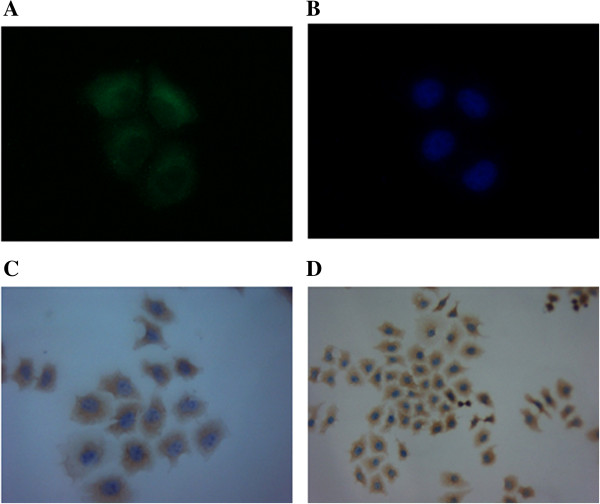
**Expression of stathmin1 in human gastric cancer cells line MKN-45. (A)** Immunofluorescences analysis of MKN-45 cells showing stathmin1 overexpression in MKN-45 cells and, **(B)** DAPI stained cells (blue). **(C)** &**(D)** Immunocytochemical analysis shows strong immunoreactivity of stathmin1 in the cytoplasm of MKN-45. The data are representative from three independent experiments with similar results.

**Figure 2:**
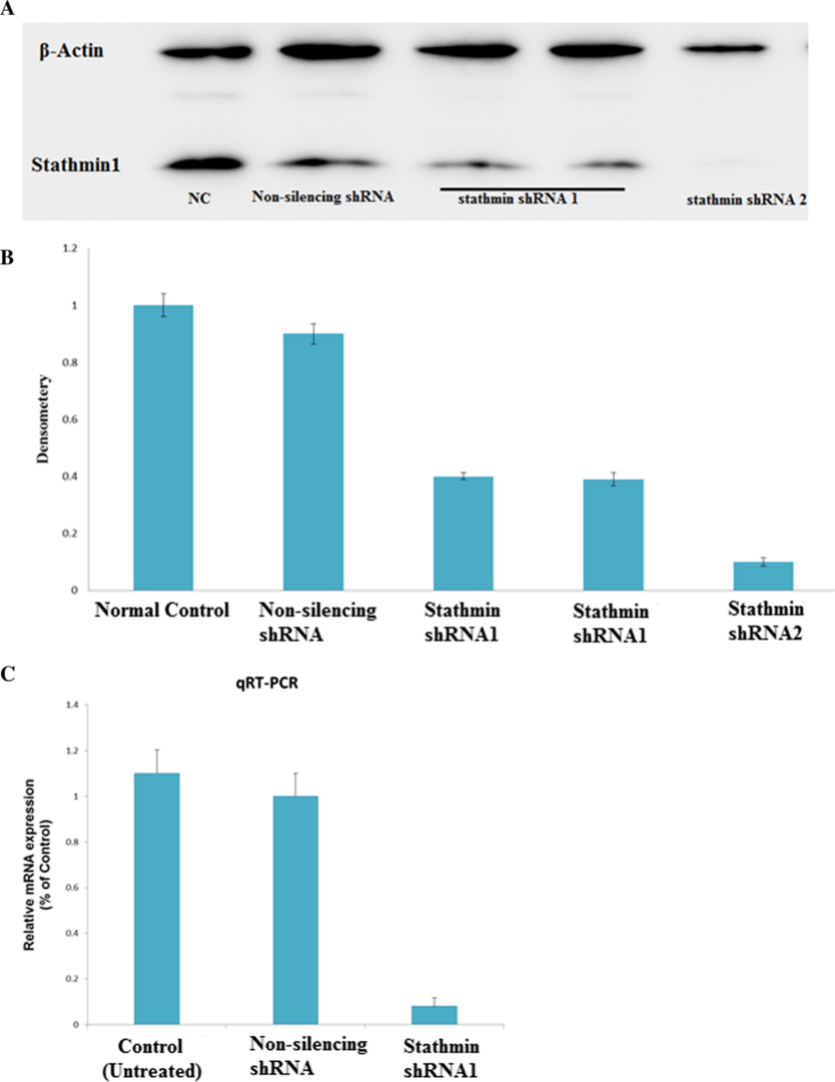
**Western blot Assay and qRT-PCR analysis of gastric cancer-derived cell lines MKN-45 with Non-silencing shRNA and stathmin shRNA knockdown. (A)** MKN-45 cells were transfected with two different Stathmin1-specific shRNA or with a Non-silencing shRNA. Forty-eight hours after transfection, the cells were analyzed by western blotting for stathmin1 **(B)** Densitometry analysis of Western blots from three independent experiments, respectively, showing that stathmin1 shRNA specifically knockdown stathmin1 expression in gastric cancer cell lines **(C)** Bar graph showing qRT–PCR analysis of cells transfected with the shRNA2 using primers specific for stathmin1 or β-actin mRNA. stathmin 1 shRNA2 significantly down-regulate stathmin mRNA level as compared with Control or transduction with Non-silenecing shRNA in MKN-45 cell lines. Data are expressed as percentage change (Means ± S.D.) compared with controls and represent four independent experiments. (*P* < 0.05 *vs* Non-silencing shRNA, one-way analysis of variance (ANOVA) followed by Tukey’s multiple comparion).

We assessed the stathmin1 gene expression in gastric cancer derived cell lines MKN-45 by immunofluorescence (IF) analysis as shown in the (Figure 1A, B) and immunocytochemistry (ICC) (Figure 1C, D). Strong immunoreactivity of stathmin1 protein was detected in the cytoplasm of MKN-45 cells. We also evaluated the state of stathmin1 protein expression in MKN-45 cell line by Western blot analysis (Figure 2A).

### Transfection & transduction efficiency

Successful packing of lentivirus was done in HEK 293 T. To determines the efficacies of viral vectors, viral supernatants prepared from either non-silencing-shRNA (control) or stathmin1shRNA were added to gastric cancer cells. Transfection efficiencies were determined by fluorescence microscopy. To evaluate the silencing efficiency, qRT-PCR and western blotting analysis were performed.

### Lentivirus-mediated RNAi efficiently suppressed stathmin1 protein and mRNA expression in MKN-45 cells

Stable expression of stathmin1 shRNAs specifically knocks down the stathmin1 expression and activity in MKN-45 gastric cells. MKN-45 cells express high levels of stathmin1, and are aggressively growing and metastatic. To investigate the role of stathmin1 in MKN-45 cell’s growth and metastasis, we constructed lentivirus vector with stathmin1 shRNA and infected MKN-45 cells. After viral infection, more than 95% of the cells were GFP-positive, indicating a high efficiency of shRNA delivery. Stathmin1 shRNA2 more efficiently knockdown protein expression in MKN-45 cell line as compared to normal control and non-silencing group (p < 0.05) (Figure 2A, B). Stathmin1 shRNA2 also efficiently suppressed the stathmin1 mRNA level conformed by qRT-PCR. We selected stathmin1 shRNA2 for further study (Figure 2C).

### Stable knockdown of oncogenic stathmin1 by lenti-shRNA significantly inhibited gastric cell growth in vitro

**Figure 3:**
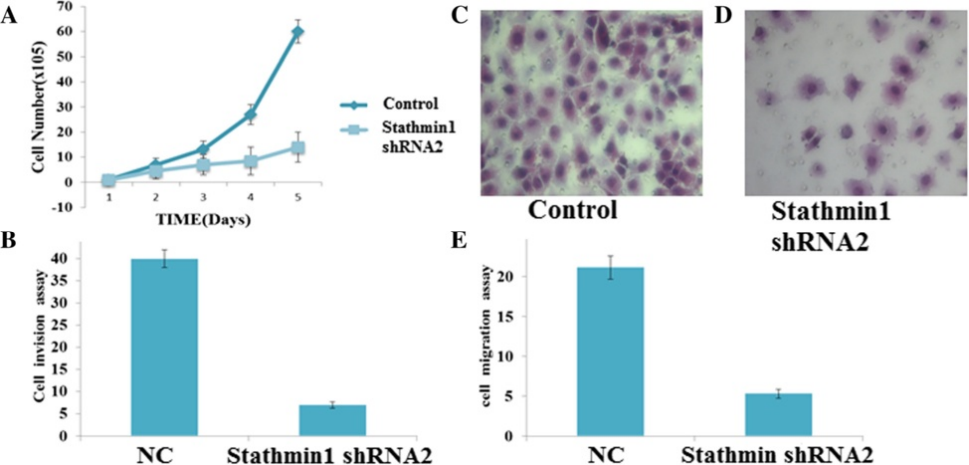
**Knockdown of stathmin 1 inhibited gastric cancer cell proliferation, invasion and migration. (A)** Proliferation curve of a MKN-45 cell line following transfection with specific stathmin1 shRNA was determined using CCK-8 assay, 96 hours after transfection. Values are normalized against the Non-silencing shRNA-negative control. **(B)** Cell invasion was evaluated in the Matrigel invasion. Data are expressed as percentage change (Means ± S.D.) compared with controls and represent four independent experiments. (*P* < 0.05 *vs* Non-silencing shRNA, one-way analysis of variance (ANOVA) followed by Tukey’s multiple comparion). **(C)** &**(D)** Cell migration was evaluated in the Boyden migration assay two days after MKN-45 cells were transfected with Non-silencing short hairpin RNA (shRNA) or stathmin1 shRNA. **(E)** Shows that untreated MKN-45 were significantly migrated across the wells as compared with stathmin1 shRNA (*P* < 0.05).

Frequent up-regulation of stathmin1 mRNA and protein in gastric cancer cell suggested a potential oncogenic role of this gene. Stathmin1 targeting impaired proliferation of MKN-45 cell lines. To investigate the possible anti-proliferative effects of stathmin1 knockdown in vitro, a CCK-8 assay was performed and a cell growth curve was generated. Stathmin1 shRNA2 transduced MKN-45 cells showed significantly reduced viability relative to control group. (Figure 3A). Stathmin1 knockdown inhibited the proliferation of MKN-45 cells in vitro, indicating that the expression of stathmin1 affects the growth of gastric cells.

### Stable knockdown of stathmin1 expression inhibits invasion and migration of MKN-cells

To evaluate the function of stathmin1sh RNA on gastric cancer cell invasion, Matrigel invasion chambers were utilized. Inhibited stathmin1 expression led to a significantly decreased invasive ability of gastric cancer cells (Figure 3B).

Cell migration was evaluated in the Boyden migration assay two days after MKN-45 cells were transfected with stathmin1 shRNA2 or transfected (control) group. Cells with motile capacity could migrate through the pores of the Trans well filters because of the attraction to 10% FBS in the lower chamber. Cells transfected with stathmin1 shRNA2 displayed less migration compared with non-transfected control cells, three-fold increases in the number of migrating cells (Figure 3C, D, E). The introduction of stathmin1shRN into cells, however, markedly decreased the cell migration, in comparison to non-transfected cells.

### In vivo studies of gastric cancer xenograft tumor models in nude mice

**Figure 4:**
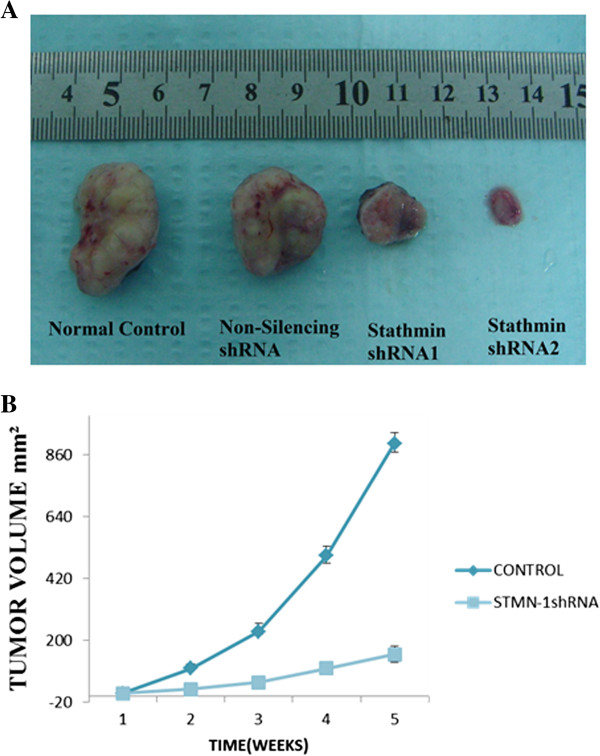
**Silencing effect of stathmin shRNA on gastric cancer xenograft in nude mice.** MKN-45 cells were transfected with stathmin1 (STMN1) shRNA or, Non-silencing short hairpin RNA (shRNA) and nude mice were inoculated subcutaneously with 2 × 10^6^ cells at two sites per mouse. The tumor mass (xenograft) volume was measured every week from week 3 to week 7. Data are expressed as the (Means ± S.D) and represent six independent experiments. (P < 0.05 vs Non-silencing shRNA, one-way analysis of variance (ANOVA) followed by Tukey’s multiple comparison). **(A)** Photograph of xenografts dissected from nude mice after 7 weeks subcutaneous inoculation showing suppression growth of cancer cells transfected with stathmin1 shRNA2 rather than non-treated control group. **(B)** Tumor growth curve showing a significant growth tendency in non-infected group (control) as compared to the stathmin shRNA2 transfected group (p < 0.05).

To further evaluate the effects of reduced stathmin1 expression on the tumorigenic phenotype and in particular its contribution to in vivo tumor growth. MKN-45 cells infected with non-silencing- shRNA and stathmin1 shRNA2 or untreated were injected into mice, stathmin shRNA2 transfected grew rapidly as compared to negative control (p < 0.05) (Figure 4A, B). These results demonstrate that in vivo tumor growth was inhibited by shRNA-mediated knockdown of stathmin1 expression in MKN-45 cell lines.

## Discussion

The cell cycle is a tightly regulated process, with each step carefully monitored by a specific group of proteins that act as checkpoints for proper cell division. The balance between these key checkpoints proteins is critical for the progression through each step of the cell cycle to occur. Stathmin 1 (STMN-1), also known as p17, p18, p19, 19 K, metablastin, oncoprotein 18, LAP 18 and Op18, is a 19 kDa cytosolic protein. Its function as an important regulatory protein of microtubule dynamics has been well characterized [7]. It has been reported that stathmin1 is overexpressed in many human malignancies, such as leukemia, lymphoma, neuroblastoma, ovarian, prostatic, breast and lung cancers [18] and the modulation of its expression correlates with invasion and metastasis. The protein expression of stathmin1 has been explored and found to correlate to clinicopathologic factors and poor prognosis in several cancers in different tissues such as brain [19], oral mucosa [20], breast [12,21,22], urothelial [23] as well as ovarian [24], melanoma [25] and uterine cervix [26]. Recently some reports showed that stathmin-1 is related to lymph node metastasis either [27]. Our study on esophageal squamous cell cancer revealed that stathmin1 is a predictor of survival in stage IIA esophageal squamous cell carcinoma [16].

Stathmin1 has been implicated in G1-S checkpoint control of cell cycle progression by influencing the dynamics of microtubule formation and progression of the cell cycle [28]. In human cancers, Stathmin1 overexpression is associated with increased malignancy, metastasis formation and decreased patient overall survival suggesting that Stathmin1 could serve as a molecular marker to identify patients with more aggressive disease [29]. Recent studies have shown that inhibition of STMN-1 expression in malignant cells interferes with their orderly progression through the cell cycle and abrogates their transformed phenotype [30]. Thus, Stathmin1 provides an attractive molecular target for disrupting the mitotic apparatus and arresting the growth of malignant cells.

In less than a decade after discovery, RNA interference-mediated gene silencing is already being tested as potential therapy in clinical trials for a number of diseases. Lentiviral vectors provide a means to express short hairpin RNA (shRNA) to induce stable and long-term gene silencing in both dividing and non-dividing cells [31]. Lentivirus-mediated RNAi also efficiently knockdown cancer gene expression in lung cancer [32,33] also decreased tumorigenicity in human oral carcinoma cells [34]. RNA interference using small inhibitory RNA (siRNA) has become a powerful tool to downregulate mRNA levels by cellular nucleases that become activated when a sequence homology between the siRNA and a respective mRNA molecule is detected. Therefore siRNA can be used to silence genes involved in the pathogenesis of various diseases associated with a known genetic background. RNAi technology facilitates a posttranscriptional gene silencing via short double-stranded RNA which is capable of binding to a specific mRNA sequence and down regulating the gene expression [35]. However, one of the major barriers to realizing the full medicinal potential of RNAi is the difficulty of delivering it in vivo and the knockdown effect of regular synthesized siRNA only lasts for a short time and does not allow the stable inhibition of target gene function. Lentivirus-mediated delivery of shRNA is more efficient and effective over other systems as it will allow for gene silencing in non-dividing cells [36]. Some studies showed that shRNA are potential therapeutic for gene therapy [37]. RNA interference also provides a paradigm to develop strategies to inactivate essential genes promoting neoplastic growth [38]. Recent advances in gene therapy approaches also indicate that systemic treatment with lentiviral shRNA vectors may be feasible. However, some form of targeted delivery approach may be needed for this. The lentiviral vectors for shRNA expression were used in our system. This approach allows for the stable suppression of target gene expression both in cell culture conditions and in animals. We found that stable knockdown of statmin1 led to reduced proliferation rates (Figure 3A) and migration in vitro (Figure 3C, D) and decreased in vivo tumorigenicity (Figure 4A, B).

Stathmin1 expression in oral cancer found to be correlated with tumor progression and poor prognosis [20]. STMN-1 increases the proliferation rate of leukemia cells [39]. We evaluated the efficacy of stathmin shRNA on the migration and proliferation of gastric cancer cells. Our findings showed that stathmin shRNA transfection in gastric cancer cells dramatically suppressed proliferation and inhibit migration ability.

To validate effectiveness of down regulation of stathmin1 expression, we performed in vivo studies in nude mice; tumor growth was greatly slowed down in stathmin1 shRNA transfected cells while non-silencing shRNA infected xenograft and control graft grew aggressively in mice model. In future, lentiviral-based RNAi, because of its potency, could be utilized as an effective strategy for cancer therapy. Additionally, the blockage of proliferation in gastric cell lines and the inhibition of tumorigenesis in nude mice support the effectiveness of this strategy. Of the new anticancer drugs approved by the U.S. Food and Drug Administration (FDA) since 2000, fifteen have been targeted drug therapies, compared with only five traditional chemotherapeutic agents [40]. Since the stathmin1 is critical for growth and the invasive behavior of gastric cancer, silencing of the stathmin1 gene with RNAi may provide a novel strategy for investigation of the role of stathmin1 gene in the invasion of human gastric cancers.

## Conclusion

Our findings provide compelling evidence of a novel role for the stathmin1 gene expression in pathogenesis of gastric cancer. We have shown that statmin1 gene expression is crucial for cell proliferation, migration in vitro and tumorigenicity in nude mice. Lentivirus-mediated delivery of stathmin1 shRNA has effectively down regulated the expression of stathmin1 gene on mRNA and protein levels. Moreover, silencing of stathmin1 has led to the significant decrease in proliferation and inhibition of migration of MKN-45 cells in vitro and slowdown the tumor growth in nude mice. Therefore, these results suggest that the proliferation and cell migration of MKN-45 cells could be regulated by silencing stathmin1 gene which is a promising gene therapeutic method to treat gastric cancer.

## Competing interests

The authors declare that they have no competing interests.

## Authors’ contributions

JA conceived the study and was involved in its design, performed the RNAi transfection experiments in vitro and in vivo, acquisition of data, statistical analysis and drafted the manuscript. JA and ZPZ performed laboratory experiments, analyzed and interpreted the data. MMB and JA culture the cells and established xenograft tumor model in mice. ZW supervised the study, helped to analyze the data, participated in the statistical analysis, and revising it critically for important intellectual content. All authors read and approved the final manuscript. No writing assistance was used in the production of the manuscript.
